# 9-(2,4-Dinitro­phen­yl)-3,3,6,6-tetra­methyl-3,4,5,6,7,9-hexa­hydro-1*H*-xanthene-1,8(2*H*)-dione

**DOI:** 10.1107/S1600536813001384

**Published:** 2013-01-23

**Authors:** N. Sureshbabu, V. Sughanya

**Affiliations:** a Department of Chemistry, Annamalai University, Annamalai Nagar 608 002, Tamil Nadu, India

## Abstract

In the title compound, C_23_H_24_N_2_O_7_, the central 4*H*-pyran ring adopts a flattened boat conformation, whereas both cyclo­hexenone rings adopt envelope conformations, the C atom bearing the dimethyl substituent being the flap atom in each case. The mean and maximum deviation of the pyran ring are 0.0379 (4) and 0.0605 (3) Å. The mean plane of the pyran ring and the dinitro­benzene ring make a dihedral angle of 85.88 (2)°.

## Related literature

For the synthesis of xanthenes, see: Vanag & Stankevich (1960 ▶); Hilderbrand & Weissleder (2007 ▶). For their pharmaceutical properties, see: Dimmock *et al.* (1988 ▶); Lambert *et al.* (1997 ▶); Poupelin *et al.* (1978 ▶); Hideo (1981 ▶); Selvanayagam *et al.* (1996 ▶). For bond-length data, see: Allen *et al.* (1987 ▶). For related structures, see: Odabaşoğlu *et al.* (2008 ▶); Reddy *et al.* (2009 ▶); Mehdi *et al.* (2011 ▶); Sughanya & Sureshbabu (2012 ▶). For ring conformation analysis, see: Cremer & Pople (1975 ▶).
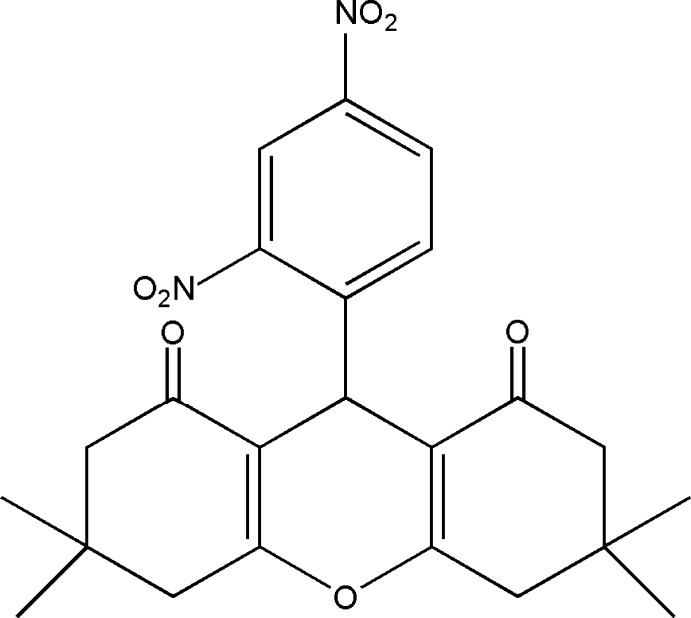


## Experimental

### 

#### Crystal data

C_23_H_24_N_2_O_7_
*M* *_r_* = 440.44
Monoclinic, 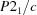

*a* = 9.7733 (3) Å
*b* = 19.6193 (5) Å
*c* = 11.7922 (3) Å
β = 109.603 (1)°
*V* = 2130.04 (10) Å^3^
*Z* = 4
Mo *K*α radiation
μ = 0.10 mm^−1^
*T* = 296 K
0.35 × 0.30 × 0.25 mm

#### Data collection

Bruker Kappa APEXII CCD diffractometer
Absorption correction: multi-scan (*SADABS*; Bruker, 2004 ▶) *T*_min_ = 0.905, *T*_max_ = 0.975
29785 measured reflections
7327 independent reflections
4793 reflections with *I* > 2σ(*I*)
*R*_int_ = 0.031

#### Refinement

*R*[*F* ^2^ > 2σ(*F* ^2^)] = 0.052
*wR*(*F* ^2^) = 0.159
*S* = 1.03
7327 reflections
289 parameters
H-atom parameters constrained
Δρ_max_ = 0.38 e Å^−3^
Δρ_min_ = −0.30 e Å^−3^

### 

Data collection: *APEX2* (Bruker, 2004 ▶); cell refinement: *APEX2* and *SAINT* (Bruker, 2004 ▶); data reduction: *SAINT* and *XPREP* (Bruker, 2004 ▶); program(s) used to solve structure: *SIR92* (Altomare *et al.*, 1993 ▶); program(s) used to refine structure: *SHELXL97* (Sheldrick, 2008 ▶); molecular graphics: *ORTEP-3 for Windows* (Farrugia, 2012 ▶) and *Mercury* (Macrae *et al.*, 2008 ▶); software used to prepare material for publication: *SHELXL97*.

## Supplementary Material

Crystal structure: contains datablock(s) global, I. DOI: 10.1107/S1600536813001384/im2418sup1.cif

Structure factors: contains datablock(s) I. DOI: 10.1107/S1600536813001384/im2418Isup2.hkl

Supplementary material file. DOI: 10.1107/S1600536813001384/im2418Isup3.cml

Additional supplementary materials:  crystallographic information; 3D view; checkCIF report

## Figures and Tables

**Fig. 1.**
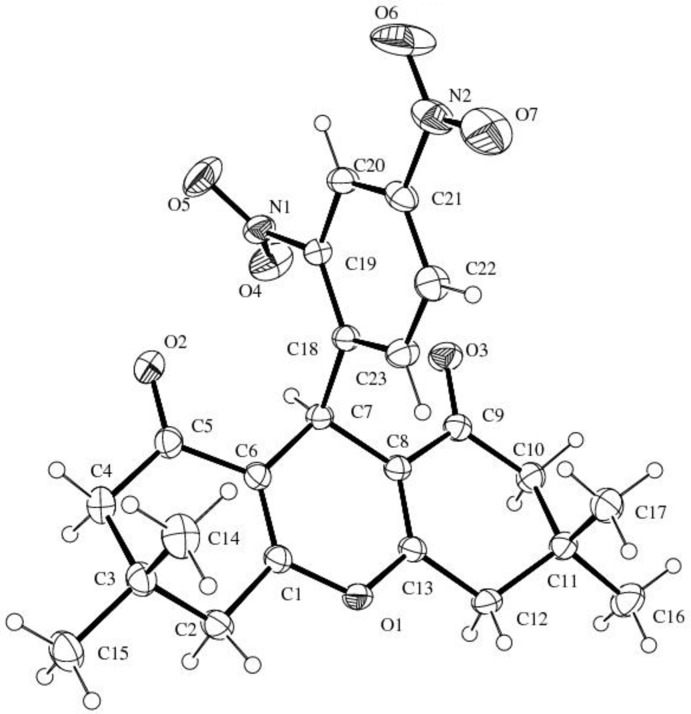
A view of the structure of title compound, showing the atom numbering scheme. Displacement ellipsoids are drawn at the 50% probability level.

## supplementary crystallographic information

### Comment

Xanthene is the parent compound of a number of naturally occurring substances and some synthetic dyes. Xanthene derivatives are used as dyes (Hilderbrand & Weissleder, 2007), possess biological properties like antibacterial, antiviral and anti-inflammatory (Dimmock *et al.*, 1988) activities and are used in medicine. Ehretianone, a quinonoid xanthene was reported to possess antisnake venom activity (Selvanayagam *et al.*, 1996; Lambert *et al.*, 1997; Poupelin *et al.*, 1978; Hideo, 1981).

The central pyran B (O1/C1/C6/C7/C8/C13) ring almost planar with a mean deviation from the mean plane of 0.0379 (4) Å and a maximum deviation of 0.061 (3) Å for C7. O1 and C7 are moved out of this mean plane towards the direction which means that the ring may also be described as a highly flattened boat conformation. The rings A (C8—C13), B (O1/C1/C6/C7/C8/C13) and C (C1—C6) show total puckering amplitudes Q(T) of 0.4602 (15) Å, 0.0988 (2) Å, 0.4479 (16) Å, respectively. The cyclohexenone rings A and C both adopt envelope conformations, whereas the central B ring adopts a flattened boat conformation. This can be rationalized by the respective puckering parameters (Cremer & Pople, 1975) φ = 177.6 (2)° and θ = 53.65 (2)° for A, φ = 179.0 (8)° and θ = 84.7 (2)° for B, φ = -54.5 (12)° and θ = 126.82 (2)° for C, respectively. The planar phenyl substituent and the central pyran ring form a dihedral angle of 85.88 (2)°. In the title compound, bond lengths (Allen *et al.*, 1987) and angles are generally within normal ranges. In the pyran ring C1—C6 and C8—C13 are double bonds in nature (C1—C6 1.333 (8) Å and C8—C13 1.334 (2) Å), as indicated by the bond distances. The C1—C6—C5 (118.81 (12)°) and C13—C8—C9 (118.70 (2)°) angles are almost identical. In this conformation C3 and C11 must be described as flap atoms being situated out of the plane of the ring with deviations of 0.316 (2) Å and 0.325 (2) Å, respectively. The observed carbonyl bond lengths C5—O2 = 1.223 (2) Å and C9—O3 = 1.216 (2) Å are also normal.

### Experimental

Following a literature method (Vanag & Stankevich, 1960) a mixture of 2,4-dinitrobenzaldehyde (0.588 g, 3 m mol) and 5,5-dimethylcyclohexane-1,3-dione (0.84 g, 6 m mol) was dissolved in 25 ml of ethanol in a 100 ml round bottomed flask. To this solution about 15 drops of concentrated hydrochloric acid were added and the content was refluxed for 30 minutes. The reaction was monitored by TLC. After completion of the reaction, the reaction mixture was poured into crushed ice and stirred well. The formed precipitate was filtered and dried. The yellow crystal used for data collection was obtained by crystallization from ethanol at room temperature,(m.p.446 K, yield: 86%).

### Refinement

Hydrogen atoms were fixed in calculated positions and allowed to ride on their parent atom with distances of d(C–H) = 0.96 Å (for CH_3_) with *U*_iso_(H) = 1.5*U*_eq_(C), d(C–H) = 0.97 Å (for CH_2_) with *U*_iso_(H) = 1.2*U*_eq_(C), d(C–H) = 0.98 Å (for CH) with *U*_iso_(H) = 1.2*U*_eq_(C) and d(C–H) = 0.93 Å (for aromatic CH) with *U*_iso_(H) = 1.2*U*_eq_(C).

### Crystal data

**Table:**

C_23_H_24_N_2_O_7_	*F*(000) = 928
*M**_r_* = 440.44	*D*_x_ = 1.373 Mg m^−^^3^
Monoclinic, *P*2_1_/*c*	Melting point: 446 K
Hall symbol: -P 2ybc	Mo *K*α radiation, λ = 0.71073 Å
*a* = 9.7733 (3) Å	Cell parameters from 8512 reflections
*b* = 19.6193 (5) Å	θ = 2.2–31.1°
*c* = 11.7922 (3) Å	µ = 0.10 mm^−^^1^
β = 109.603 (1)°	*T* = 296 K
*V* = 2130.04 (10) Å^3^	Block, yellow
*Z* = 4	0.35 × 0.30 × 0.25 mm

### Data collection

**Table:**

Bruker Kappa APEXII CCD diffractometer	7327 independent reflections
Radiation source: fine-focus sealed tube	4793 reflections with *I* > 2σ(*I*)
Graphite monochromator	*R*_int_ = 0.031
ω and φ scan	θ_max_ = 32.0°, θ_min_ = 2.1°
Absorption correction: multi-scan (*SADABS*; Bruker, 2004)	*h* = −14→14
*T*_min_ = 0.905, *T*_max_ = 0.975	*k* = −29→27
29785 measured reflections	*l* = −15→17

### Refinement

**Table:**

Refinement on *F*^2^	Primary atom site location: structure-invariant direct methods
Least-squares matrix: full	Secondary atom site location: difference Fourier map
*R*[*F*^2^ > 2σ(*F*^2^)] = 0.052	Hydrogen site location: inferred from neighbouring sites
*wR*(*F*^2^) = 0.159	H-atom parameters constrained
*S* = 1.03	*w* = 1/[σ^2^(*F*_o_^2^) + (0.0777*P*)^2^ + 0.3517*P*] where *P* = (*F*_o_^2^ + 2*F*_c_^2^)/3
7327 reflections	(Δ/σ)_max_ < 0.001
289 parameters	Δρ_max_ = 0.38 e Å^−^^3^
0 restraints	Δρ_min_ = −0.30 e Å^−^^3^

### Special details

**Table:**

Geometry. All e.s.d.'s (except the e.s.d. in the dihedral angle between two l.s. planes) are estimated using the full covariance matrix. The cell e.s.d.'s are taken into account individually in the estimation of e.s.d.'s in distances, angles and torsion angles; correlations between e.s.d.'s in cell parameters are only used when they are defined by crystal symmetry. An approximate (isotropic) treatment of cell e.s.d.'s is used for estimating e.s.d.'s involving l.s. planes.
Refinement. Refinement of *F*^2^ against ALL reflections. The weighted *R*-factor *wR* and goodness of fit *S* are based on *F*^2^, conventional *R*-factors *R* are based on *F*, with *F* set to zero for negative *F*^2^. The threshold expression of *F*^2^ > σ(*F*^2^) is used only for calculating *R*-factors(gt) *etc*. and is not relevant to the choice of reflections for refinement. *R*-factors based on *F*^2^ are statistically about twice as large as those based on *F*, and *R*- factors based on ALL data will be even larger.

### Fractional atomic coordinates and isotropic or equivalent isotropic displacement parameters (Å^2^)

**Table:**

	*x*	*y*	*z*	*U*_iso_*/*U*_eq_	
C1	0.51973 (13)	0.15131 (7)	0.58305 (11)	0.0339 (3)	
C2	0.41933 (15)	0.19984 (8)	0.61245 (13)	0.0434 (3)	
H2A	0.4760	0.2353	0.6648	0.052*	
H2B	0.3659	0.1758	0.6561	0.052*	
C3	0.31152 (14)	0.23285 (8)	0.50052 (13)	0.0397 (3)	
C4	0.24534 (14)	0.17645 (8)	0.40933 (14)	0.0432 (3)	
H4A	0.1827	0.1488	0.4393	0.052*	
H4B	0.1851	0.1973	0.3347	0.052*	
C5	0.35407 (13)	0.13059 (7)	0.38268 (12)	0.0372 (3)	
C6	0.49306 (12)	0.11891 (7)	0.47847 (11)	0.0325 (3)	
C7	0.59949 (13)	0.06997 (6)	0.45403 (10)	0.0303 (2)	
H7	0.5504	0.0265	0.4260	0.036*	
C8	0.72373 (13)	0.05827 (6)	0.56996 (11)	0.0309 (2)	
C9	0.83150 (13)	0.00583 (7)	0.57141 (11)	0.0338 (3)	
C10	0.95370 (15)	−0.00702 (7)	0.68746 (12)	0.0393 (3)	
H10A	1.0378	−0.0227	0.6687	0.047*	
H10B	0.9251	−0.0433	0.7308	0.047*	
C11	0.99723 (14)	0.05519 (7)	0.76956 (12)	0.0383 (3)	
C12	0.85972 (15)	0.08387 (8)	0.78692 (11)	0.0406 (3)	
H12A	0.8279	0.0530	0.8374	0.049*	
H12B	0.8822	0.1274	0.8281	0.049*	
C13	0.73989 (13)	0.09328 (7)	0.67058 (11)	0.0330 (3)	
C14	0.38929 (18)	0.28478 (9)	0.44734 (16)	0.0541 (4)	
H14A	0.4308	0.3198	0.5056	0.081*	
H14B	0.3210	0.3048	0.3766	0.081*	
H14C	0.4649	0.2624	0.4264	0.081*	
C15	0.19240 (17)	0.26822 (10)	0.53505 (17)	0.0551 (4)	
H15A	0.2344	0.3036	0.5925	0.083*	
H15B	0.1449	0.2356	0.5699	0.083*	
H15C	0.1228	0.2878	0.4645	0.083*	
C16	1.10517 (18)	0.03431 (10)	0.89168 (14)	0.0594 (5)	
H16A	1.0614	0.0007	0.9279	0.089*	
H16B	1.1313	0.0736	0.9431	0.089*	
H16C	1.1906	0.0155	0.8808	0.089*	
C17	1.06762 (17)	0.10848 (9)	0.71315 (15)	0.0509 (4)	
H17A	1.0003	0.1220	0.6365	0.076*	
H17B	1.1530	0.0896	0.7023	0.076*	
H17C	1.0940	0.1475	0.7651	0.076*	
C18	0.65475 (12)	0.09886 (6)	0.35707 (10)	0.0295 (2)	
C19	0.61732 (13)	0.07634 (7)	0.23872 (11)	0.0313 (2)	
C20	0.66867 (14)	0.10639 (7)	0.15506 (11)	0.0361 (3)	
H20	0.6414	0.0904	0.0764	0.043*	
C21	0.76167 (14)	0.16090 (7)	0.19241 (12)	0.0379 (3)	
C22	0.80053 (16)	0.18654 (8)	0.30670 (13)	0.0425 (3)	
H22	0.8618	0.2241	0.3294	0.051*	
C23	0.74630 (15)	0.15511 (7)	0.38759 (12)	0.0382 (3)	
H23	0.7721	0.1723	0.4655	0.046*	
N1	0.51735 (13)	0.01863 (7)	0.19194 (10)	0.0422 (3)	
N2	0.81838 (16)	0.19342 (8)	0.10531 (13)	0.0543 (4)	
O1	0.64491 (10)	0.14292 (5)	0.67914 (8)	0.0384 (2)	
O2	0.32732 (11)	0.10217 (6)	0.28536 (9)	0.0516 (3)	
O3	0.81924 (12)	−0.02696 (6)	0.48107 (9)	0.0491 (3)	
O4	0.52780 (15)	−0.03162 (6)	0.25373 (11)	0.0642 (4)	
O5	0.42986 (15)	0.02476 (8)	0.09097 (11)	0.0723 (4)	
O6	0.7810 (2)	0.17153 (9)	0.00386 (14)	0.0984 (6)	
O7	0.89653 (17)	0.24275 (8)	0.13837 (14)	0.0805 (4)	

### Atomic displacement parameters (Å^2^)

**Table:**

	*U*^11^	*U*^22^	*U*^33^	*U*^12^	*U*^13^	*U*^23^
C1	0.0318 (5)	0.0406 (7)	0.0304 (6)	0.0049 (5)	0.0120 (5)	0.0037 (5)
C2	0.0425 (7)	0.0530 (9)	0.0372 (7)	0.0145 (6)	0.0168 (6)	0.0015 (6)
C3	0.0347 (6)	0.0417 (8)	0.0427 (7)	0.0055 (5)	0.0131 (5)	0.0038 (6)
C4	0.0306 (6)	0.0467 (8)	0.0494 (8)	0.0011 (5)	0.0095 (5)	0.0013 (6)
C5	0.0308 (6)	0.0426 (7)	0.0377 (7)	−0.0029 (5)	0.0109 (5)	0.0018 (5)
C6	0.0295 (5)	0.0382 (7)	0.0311 (6)	0.0010 (5)	0.0119 (4)	0.0029 (5)
C7	0.0317 (5)	0.0334 (6)	0.0268 (5)	−0.0018 (5)	0.0109 (4)	−0.0004 (4)
C8	0.0317 (5)	0.0343 (6)	0.0279 (5)	0.0012 (5)	0.0116 (4)	0.0014 (5)
C9	0.0354 (6)	0.0329 (6)	0.0343 (6)	−0.0005 (5)	0.0133 (5)	−0.0013 (5)
C10	0.0377 (6)	0.0379 (7)	0.0400 (7)	0.0065 (5)	0.0099 (5)	−0.0002 (6)
C11	0.0346 (6)	0.0439 (8)	0.0331 (6)	0.0061 (5)	0.0069 (5)	−0.0041 (5)
C12	0.0403 (6)	0.0539 (9)	0.0263 (6)	0.0099 (6)	0.0094 (5)	−0.0011 (5)
C13	0.0328 (5)	0.0394 (7)	0.0283 (6)	0.0063 (5)	0.0125 (4)	0.0025 (5)
C14	0.0495 (8)	0.0476 (9)	0.0650 (10)	−0.0020 (7)	0.0189 (7)	0.0097 (8)
C15	0.0455 (8)	0.0576 (10)	0.0643 (10)	0.0162 (7)	0.0212 (7)	0.0023 (8)
C16	0.0495 (8)	0.0747 (12)	0.0417 (8)	0.0207 (8)	−0.0010 (7)	−0.0062 (8)
C17	0.0452 (8)	0.0524 (9)	0.0559 (9)	−0.0092 (7)	0.0180 (7)	−0.0133 (7)
C18	0.0309 (5)	0.0316 (6)	0.0270 (5)	0.0013 (4)	0.0108 (4)	0.0006 (4)
C19	0.0321 (5)	0.0334 (6)	0.0281 (6)	0.0002 (5)	0.0098 (4)	−0.0014 (5)
C20	0.0403 (6)	0.0426 (7)	0.0272 (6)	0.0066 (5)	0.0137 (5)	0.0023 (5)
C21	0.0406 (6)	0.0399 (7)	0.0399 (7)	0.0055 (5)	0.0222 (5)	0.0101 (5)
C22	0.0452 (7)	0.0382 (7)	0.0463 (8)	−0.0086 (6)	0.0183 (6)	0.0017 (6)
C23	0.0444 (7)	0.0381 (7)	0.0328 (6)	−0.0073 (6)	0.0138 (5)	−0.0034 (5)
N1	0.0436 (6)	0.0483 (7)	0.0337 (6)	−0.0087 (5)	0.0115 (5)	−0.0092 (5)
N2	0.0629 (8)	0.0574 (9)	0.0552 (8)	0.0032 (7)	0.0367 (7)	0.0144 (7)
O1	0.0376 (5)	0.0481 (6)	0.0284 (4)	0.0115 (4)	0.0096 (4)	−0.0031 (4)
O2	0.0387 (5)	0.0704 (8)	0.0398 (6)	0.0021 (5)	0.0052 (4)	−0.0092 (5)
O3	0.0528 (6)	0.0513 (6)	0.0419 (6)	0.0084 (5)	0.0141 (5)	−0.0121 (5)
O4	0.0859 (9)	0.0470 (7)	0.0540 (7)	−0.0244 (6)	0.0157 (6)	−0.0048 (5)
O5	0.0660 (8)	0.0897 (10)	0.0424 (6)	−0.0206 (7)	−0.0067 (6)	−0.0088 (6)
O6	0.1521 (16)	0.1066 (13)	0.0657 (9)	−0.0304 (11)	0.0749 (11)	−0.0055 (9)
O7	0.0924 (10)	0.0799 (10)	0.0826 (10)	−0.0268 (8)	0.0471 (8)	0.0168 (8)

### Geometric parameters (Å, º)

**Table:**

C1—C6	1.3331 (18)	C12—H12B	0.9700
C1—O1	1.3702 (15)	C13—O1	1.3723 (15)
C1—C2	1.4892 (18)	C14—H14A	0.9600
C2—C3	1.5293 (19)	C14—H14B	0.9600
C2—H2A	0.9700	C14—H14C	0.9600
C2—H2B	0.9700	C15—H15A	0.9600
C3—C15	1.523 (2)	C15—H15B	0.9600
C3—C14	1.526 (2)	C15—H15C	0.9600
C3—C4	1.527 (2)	C16—H16A	0.9600
C4—C5	1.504 (2)	C16—H16B	0.9600
C4—H4A	0.9700	C16—H16C	0.9600
C4—H4B	0.9700	C17—H17A	0.9600
C5—O2	1.2226 (17)	C17—H17B	0.9600
C5—C6	1.4643 (17)	C17—H17C	0.9600
C6—C7	1.5127 (17)	C18—C23	1.3899 (18)
C7—C8	1.5107 (16)	C18—C19	1.3908 (16)
C7—C18	1.5279 (17)	C19—C20	1.3801 (18)
C7—H7	0.9800	C19—N1	1.4762 (17)
C8—C13	1.3338 (17)	C20—C21	1.377 (2)
C8—C9	1.4683 (17)	C20—H20	0.9300
C9—O3	1.2155 (15)	C21—C22	1.368 (2)
C9—C10	1.5054 (18)	C21—N2	1.4667 (18)
C10—C11	1.5270 (19)	C22—C23	1.3820 (19)
C10—H10A	0.9700	C22—H22	0.9300
C10—H10B	0.9700	C23—H23	0.9300
C11—C17	1.522 (2)	N1—O4	1.2096 (17)
C11—C16	1.5280 (19)	N1—O5	1.2166 (16)
C11—C12	1.5327 (18)	N2—O6	1.207 (2)
C12—C13	1.4864 (17)	N2—O7	1.213 (2)
C12—H12A	0.9700		
			
C6—C1—O1	123.43 (11)	C11—C12—H12B	109.2
C6—C1—C2	125.49 (11)	H12A—C12—H12B	107.9
O1—C1—C2	111.07 (11)	C8—C13—O1	123.36 (11)
C1—C2—C3	112.76 (11)	C8—C13—C12	125.35 (11)
C1—C2—H2A	109.0	O1—C13—C12	111.29 (11)
C3—C2—H2A	109.0	C3—C14—H14A	109.5
C1—C2—H2B	109.0	C3—C14—H14B	109.5
C3—C2—H2B	109.0	H14A—C14—H14B	109.5
H2A—C2—H2B	107.8	C3—C14—H14C	109.5
C15—C3—C14	109.66 (13)	H14A—C14—H14C	109.5
C15—C3—C4	109.71 (12)	H14B—C14—H14C	109.5
C14—C3—C4	110.24 (13)	C3—C15—H15A	109.5
C15—C3—C2	109.23 (12)	C3—C15—H15B	109.5
C14—C3—C2	110.09 (12)	H15A—C15—H15B	109.5
C4—C3—C2	107.89 (12)	C3—C15—H15C	109.5
C5—C4—C3	114.75 (11)	H15A—C15—H15C	109.5
C5—C4—H4A	108.6	H15B—C15—H15C	109.5
C3—C4—H4A	108.6	C11—C16—H16A	109.5
C5—C4—H4B	108.6	C11—C16—H16B	109.5
C3—C4—H4B	108.6	H16A—C16—H16B	109.5
H4A—C4—H4B	107.6	C11—C16—H16C	109.5
O2—C5—C6	120.23 (12)	H16A—C16—H16C	109.5
O2—C5—C4	121.70 (12)	H16B—C16—H16C	109.5
C6—C5—C4	118.04 (12)	C11—C17—H17A	109.5
C1—C6—C5	118.81 (12)	C11—C17—H17B	109.5
C1—C6—C7	123.05 (11)	H17A—C17—H17B	109.5
C5—C6—C7	118.14 (11)	C11—C17—H17C	109.5
C8—C7—C6	108.62 (10)	H17A—C17—H17C	109.5
C8—C7—C18	110.80 (10)	H17B—C17—H17C	109.5
C6—C7—C18	110.21 (10)	C23—C18—C19	116.22 (11)
C8—C7—H7	109.1	C23—C18—C7	117.34 (11)
C6—C7—H7	109.1	C19—C18—C7	126.38 (11)
C18—C7—H7	109.1	C20—C19—C18	123.04 (12)
C13—C8—C9	118.70 (11)	C20—C19—N1	114.48 (11)
C13—C8—C7	123.10 (11)	C18—C19—N1	122.48 (11)
C9—C8—C7	118.21 (11)	C21—C20—C19	117.55 (12)
O3—C9—C8	120.08 (12)	C21—C20—H20	121.2
O3—C9—C10	121.53 (12)	C19—C20—H20	121.2
C8—C9—C10	118.37 (11)	C22—C21—C20	122.43 (12)
C9—C10—C11	114.19 (11)	C22—C21—N2	119.00 (13)
C9—C10—H10A	108.7	C20—C21—N2	118.56 (13)
C11—C10—H10A	108.7	C21—C22—C23	118.17 (13)
C9—C10—H10B	108.7	C21—C22—H22	120.9
C11—C10—H10B	108.7	C23—C22—H22	120.9
H10A—C10—H10B	107.6	C22—C23—C18	122.56 (12)
C17—C11—C10	110.04 (12)	C22—C23—H23	118.7
C17—C11—C16	109.01 (13)	C18—C23—H23	118.7
C10—C11—C16	109.91 (12)	O4—N1—O5	124.03 (13)
C17—C11—C12	110.57 (12)	O4—N1—C19	119.24 (11)
C10—C11—C12	107.93 (11)	O5—N1—C19	116.72 (13)
C16—C11—C12	109.37 (11)	O6—N2—O7	123.44 (15)
C13—C12—C11	112.15 (11)	O6—N2—C21	118.66 (15)
C13—C12—H12A	109.2	O7—N2—C21	117.84 (15)
C11—C12—H12A	109.2	C1—O1—C13	117.56 (10)
C13—C12—H12B	109.2		
			
C6—C1—C2—C3	−24.1 (2)	C16—C11—C12—C13	−169.45 (14)
O1—C1—C2—C3	156.81 (12)	C9—C8—C13—O1	−179.49 (11)
C1—C2—C3—C15	166.99 (13)	C7—C8—C13—O1	0.5 (2)
C1—C2—C3—C14	−72.55 (17)	C9—C8—C13—C12	−0.1 (2)
C1—C2—C3—C4	47.79 (16)	C7—C8—C13—C12	179.97 (12)
C15—C3—C4—C5	−170.89 (13)	C11—C12—C13—C8	26.1 (2)
C14—C3—C4—C5	68.26 (16)	C11—C12—C13—O1	−154.43 (12)
C2—C3—C4—C5	−51.99 (16)	C8—C7—C18—C23	−50.45 (15)
C3—C4—C5—O2	−151.35 (14)	C6—C7—C18—C23	69.81 (14)
C3—C4—C5—C6	30.51 (18)	C8—C7—C18—C19	132.67 (13)
O1—C1—C6—C5	178.65 (12)	C6—C7—C18—C19	−107.07 (14)
C2—C1—C6—C5	−0.3 (2)	C23—C18—C19—C20	1.15 (19)
O1—C1—C6—C7	−0.9 (2)	C7—C18—C19—C20	178.06 (12)
C2—C1—C6—C7	−179.87 (13)	C23—C18—C19—N1	−178.00 (12)
O2—C5—C6—C1	179.17 (13)	C7—C18—C19—N1	−1.09 (19)
C4—C5—C6—C1	−2.66 (19)	C18—C19—C20—C21	0.32 (19)
O2—C5—C6—C7	−1.26 (19)	N1—C19—C20—C21	179.54 (11)
C4—C5—C6—C7	176.91 (12)	C19—C20—C21—C22	−1.7 (2)
C1—C6—C7—C8	7.67 (17)	C19—C20—C21—N2	179.64 (12)
C5—C6—C7—C8	−171.88 (11)	C20—C21—C22—C23	1.5 (2)
C1—C6—C7—C18	−113.88 (13)	N2—C21—C22—C23	−179.84 (13)
C5—C6—C7—C18	66.57 (14)	C21—C22—C23—C18	0.1 (2)
C6—C7—C8—C13	−7.50 (17)	C19—C18—C23—C22	−1.4 (2)
C18—C7—C8—C13	113.70 (13)	C7—C18—C23—C22	−178.56 (13)
C6—C7—C8—C9	172.53 (11)	C20—C19—N1—O4	138.94 (14)
C18—C7—C8—C9	−66.27 (14)	C18—C19—N1—O4	−41.84 (19)
C13—C8—C9—O3	179.81 (13)	C20—C19—N1—O5	−39.93 (18)
C7—C8—C9—O3	−0.22 (19)	C18—C19—N1—O5	139.28 (14)
C13—C8—C9—C10	1.31 (18)	C22—C21—N2—O6	−178.31 (17)
C7—C8—C9—C10	−178.72 (11)	C20—C21—N2—O6	0.4 (2)
O3—C9—C10—C11	152.40 (13)	C22—C21—N2—O7	−1.0 (2)
C8—C9—C10—C11	−29.12 (17)	C20—C21—N2—O7	177.70 (15)
C9—C10—C11—C17	−68.38 (15)	C6—C1—O1—C13	−7.06 (19)
C9—C10—C11—C16	171.56 (12)	C2—C1—O1—C13	172.05 (12)
C9—C10—C11—C12	52.36 (16)	C8—C13—O1—C1	7.24 (19)
C17—C11—C12—C13	70.50 (16)	C12—C13—O1—C1	−172.26 (11)
C10—C11—C12—C13	−49.90 (16)		
